# Verbal autopsy: advancing science, facilitating application

**DOI:** 10.1186/1478-7954-9-18

**Published:** 2011-07-27

**Authors:** Christopher JL Murray, Alan D Lopez, Kenji Shibuya, Rafael Lozano

**Affiliations:** 1 Institute for Health Metrics and Evaluation, University of Washington, 2301 Fifth Ave, Suite 600, Seattle, WA 98121, USA; 2 University of Queensland, School of Population Health, 288 Herston Road, Herston, Queensland 4006, Australia; 3 Graduate School of Medicine, University of Tokyo, 7-3-1, Hongo, Bunkyo-ku, Tokyo, 113-0033, Japan

## Editorial

Critical information on population health is needed to inform planning, resource allocation, program implementation, monitoring, and evaluation. One of the key descriptors of a population's health is information about causes of death. Since many countries lack complete vital registration systems with medical certification of deaths, cause of death information is often missing. Verbal autopsy (VA) can be used to determine individuals' causes of death and cause-specific mortality fractions in populations without a complete vital registration system. A standard VA instrument paired with easy-to-implement and reliable analytic methods could help bridge significant gaps in information about causes of death, particularly in resource-poor settings.

A great deal of research has been conducted in the past several decades about VA and its application in the field, particularly in research settings, but some traditional methods of implementation and analysis can be costly, time-consuming, and potentially of varying quality. Verbal autopsies can now be analyzed using a much wider array of innovative techniques, most of which will be less expensive and yield higher quality results than current practice. What has been missing from the field of verbal autopsy is a collection of the most up-to-date research to help decision-makers choose the best and most cost-effective VA techniques to identify causes of death in their populations.

This thematic series of *Population Health Metrics*, "Verbal autopsy: innovations, applications, opportunities," was developed in response to this need. The research published in this thematic series emerged from the "Global Congress on Verbal Autopsy: State of the Science," held in Bali, Indonesia, in February 2011. The conference was co-sponsored by the Institute for Health Metrics and Evaluation, the University of Queensland School of Population Health, and *Population Health Metrics*.

The Congress convened the global research and policy community who currently work with VA data, or who could greatly benefit from doing so. The conference inspired vibrant discussions about critical aspects of VA, including instrument design, analysis methods, and the potential use of VA in national health information systems. By convening a wide array of participants with different perspectives, a greater exchange of ideas, collaboration, and intellectual innovation was encouraged to advance the use and understanding of VA as a mechanism for gathering valuable information about causes of death in populations. The innovative research presented at the conference has motivated the creation of a community of scientists, policymakers, and practitioners dedicated to furthering this important field of population health.

In an effort to promote and disseminate the key research breakthroughs discussed at the Global Congress, we are publishing this thematic series. After peer review, 24 papers and eight commentaries were accepted for publication. The innovations in VA detailed in these papers represent a substantial increase in knowledge about the comparative performance of various methods to assign causes of death, from applications of methods used in current practice, including physician review, to a rigorous validation of new automated methods with significant potential for future application in routine national and research data collection platforms.

We expect that this thematic series of *Population Health Metrics *will provide an opportunity for informed discussion and debate and hopefully will stimulate the widespread application of VA where it is needed. This collection of research clearly shows that automated methods for VA are more accurate, faster, and cheaper than traditional physician review. Scientific innovation has taken VA from infancy to maturity.

While methods innovation will and must continue, we hope that this thematic series will stimulate debate, increase knowledge, and facilitate application of VA in national health information systems. We believe that the global health community, including national governments, can now more confidently measure causes of death to monitor progress toward health and development goals. We urge them to seize the opportunities for improved population health measurement that are now available.

